# Monitored Anesthesia Care Using Remimazolam and Ketamine Combination for Brief Gynecological Surgeries: A Report for Four Cases

**DOI:** 10.3390/jcm12103558

**Published:** 2023-05-19

**Authors:** Soron Choi, Ganghyun Lee, Jiwook Jung, Taeyoung Lee, Sangyoong Park

**Affiliations:** Department of Anesthesiology and Pain Medicine, Dong-A University College of Medicine, Busan 49201, Republic of Korea; choisr@dau.ac.kr (S.C.); gangnuyh@naver.com (G.L.); wnr2749@gmail.com (J.J.); eggrobo1024@gmail.com (T.L.)

**Keywords:** anesthesia and analgesia, gynecologic surgical procedures, ketamine, monitored anesthesia care, remimazolam

## Abstract

Remimazolam is a benzodiazepine with rapid onset and recovery time. Ketamine provides analgesia and sedation without compromising hemodynamics. Combining both agents may provide good anesthesia and analgesia with fewer complications. We report four cases of monitored anesthesia care with a combination of remimazolam and ketamine for brief gynecological surgeries. We applied 0.5 mg/kg bolus ketamine and infused patients with remimazolam at 6 mg/kg/h for induction and 1 mg/kg/h for maintenance. Then, 25 µg of fentanyl was administered for analgesia 4 min before the procedure, and additional fentanyl was administered as needed. Remimazolam was discontinued shortly after surgery. We conducted satisfactory monitored anesthesia care with a combination of remimazolam and ketamine in all four cases.

## 1. Introduction

Remimazolam is a promising ultra-short-acting benzodiazepine with a more rapid onset of action and shorter maintenance and recovery time than midazolam, and has been frequently chosen for procedural sedation [1,2]. However, hemodynamic compromises, including hypotension and bradycardia, which require vasopressors and treatment, were also found when using remimazolam; although, they occurred much less often than with propofol [3]. Brief procedures performed in gynecology, such as hysteroscopic dilatation/curettage/biopsy (DCB) and conization, can cause significant pain and discomfort to the patients. Therefore, additional opioids for analgesia may need to be combined with remimazolam to prevent body movement from interfering with the procedure and patient satisfaction.

Ketamine, an N-methyl-D-aspartate (NMDA) receptor antagonist, provides dissociative anesthesia and analgesia with rare respiratory depression [4,5]. Ketamine stimulates the sympathetic nervous system, leading to tachycardia and increased blood pressure. Administering small doses of ketamine perioperatively can reduce opioid requirements [6]. However, its disadvantages include emergence symptoms such as agitation, hallucinations, and delirium [4]. Therefore, ketamine is often co-administered with benzodiazepines to reduce the incidence of emergence phenomena [7].

Therefore, combining remimazolam and ketamine (“kemimazolam”) may theoretically have the advantages of both drugs, compensate for each other’s disadvantages, and provide satisfactory anesthesia and analgesia. No study has previously used remimazolam and ketamine for monitored anesthesia care for gynecologic surgical procedures. Therefore, we present four cases of kemimazolam anesthesia for simple gynecological surgeries.

## 2. Case Descriptions

### 2.1. Case 1

A 27-year-old woman (weight, 59 kg; height, 160 cm) who visited our hospital with atypical uterine bleeding and a high probability of endometrial hyperplasia was scheduled to undergo hysteroscopic DCB in the operating room under monitored anesthesia care. She had no underlying diseases, and her physical status was classified as I according to the American Society of Anesthesiologists (ASA) classification. Preoperative risk evaluation was performed before surgery, including laboratory examination, chest radiography, and electrocardiography. After providing a sufficient explanation of the study and the potential side effects of the drugs to the patient, we obtained written informed consent.

On the day of the surgery, an 18-gauge intravenous catheter was inserted peripherally. Intramuscular glycopyrrolate (0.2 mg) was administered 30 min before entering the operating room to blunt the hypersalivation. Noninvasive monitoring of blood pressure (BP), heart rate (HR), respiration rate (RR), saturation of percutaneous oxygen (SpO2), and bispectral index (BIS) was performed after entering the operating room. Her initial vital signs were stable (BP, 121/72 mmHg; HR, 69 beats/min; RR, 19 breaths/min, SpO2 100%). Vital signs were monitored continuously, and BP was monitored every 5 min and additionally as needed. Supplemental oxygen was started shortly before the induction at a rate of 5 L/min via a non-rebreathing face mask. Then, 0.5 mg/kg bolus ketamine was applied, and 6 mg/kg/h remimazolam (Byfavo; Hana Pharm CO., Ltd., Seoul, Republic of Korea) was infused continuously until the patient’s modified observer assessment of alertness/sedation (MOAA/S) score decreased to <3. The patient reported no injection pain for either drug.

The target level of sedation was checked every 10 s. After 20 s, the patient’s MOAA/S score had reached 2, and the BIS value had decreased to 78. Remimazolam was infused at 1 mg/kg/h for targeting BIS values between 60 and 80 and maintaining the target level of sedation. The remimazolam administration rate was adjusted by 0.2 mg/kg/h only when a BIS score exceeding 80 and a MOAA/S score exceeding 2 are both satisfied, or the BIS score is less than 60 and the MOAA/S score is 0 when the BIS score is out of the target range. The patient’s vital signs were stable after induction, and no other adverse events occurred. We administered 25 μg of bolus fentanyl after the induction to prevent body movement in the patient during cervical dilatation at the beginning of the surgery. Then, an additional 25 μg of fentanyl was given if any signs of insufficient analgesia, such as grimace, body movement, tachycardia (HR > 100 beats/min), or a sudden rise in systolic BP (> 140 mmHg or 20% over baseline), were present. We requested the gynecologist to start the surgery 4 min after the first administration of fentanyl. The procedure began 5 min after induction, and an adduction motion in the lower extremities was observed, and the HR increased to 105 beats/min. An additional 25 μg fentanyl was administered immediately after the patient’s body movement. Then, 8 min after induction, grimace and body movement were observed again, and an additional 25 μg of fentanyl was administered. The surgery ended 15 min after the induction, and remimazolam was discontinued shortly after the end of the surgery. In total, 19.6 mg of remimazolam was administered.

Vital signs were stable throughout the procedure, and any other adverse events, including hypotension, bradycardia, and respiratory depression, were not observed. The MOAA/S score was below three throughout the procedure, and the range of BIS values was 72–78. Her MOAA/S score increased gradually after discontinuing the remimazolam infusion. The patient responded lethargically to verbal commands (MOAA/S score 4) and voluntarily moved all extremities 4 min after remimazolam discontinuation. She was discharged from the recovery room 14 min after the end of the surgery when she was fully awake, and her modified Aldrete score reached 10. There were no adverse events, including agitation, delirium, bradycardia, hypotension, or respiratory depression. The patient was totally amnestic and did not report any unsatisfactory symptoms, such as nausea/vomiting, vivid dreams, or memory of pain during and after the procedure.

### 2.2. Case 2

A 41-year-old woman (weight, 51 kg; height, 163 cm) was diagnosed with submucosal myoma (1.2 cm). Monitored anesthesia care was planned for a resectoscopic myomectomy. Her ASA score was Ⅰ, and there were no abnormal findings in the preoperative evaluation. Written informed consent was obtained. Then, intramuscular glycopyrrolate (0.2 mg) was injected 30 min before entering the operating room. Her initial vital signs were BP 105/55 mmHg, HR 86 beat/min, RR 23 breaths/min, and SpO2 99%. Supplemental oxygen was administered before induction. Then 40 s after starting kemimazolam administration, the patient’s MOAA/S score decreased to 2, and the BIS value reached 75. Injection pain was not observed. In addition, 25 µg of fentanyl was administered 4 min before the procedure.

At the beginning of the surgery, there was no body movement, grimace, or change in any vital signs. Then, 6 min into the surgery, the patient’s BIS score increased from 75 to 82, MOAA/S score increased to 3, and body movement of the upper extremity and facial grimace were observed. An additional 25 μg of fentanyl was administered immediately after movement, and the infusion rate of remimazolam was adjusted to 1.2 mg/kg/h. Afterward, the range of vital signs remained stable, with no rise or fall in BP beyond 20%, such as the BIS value (range 75 to 78). In total, 21.4 mg of remimazolam was administered for 20 min until the end of the surgery. Then, 5 min after the end of the surgery, her MOAA/S score increased to four, and she was transferred to the recovery room. No adverse events occurred, including emergence agitation, and the patient did not complain of pain or other side effects. The patient was fully awake and cooperative (modified Aldrete score 10) 11 min after discontinuing remimazolam and was discharged from the recovery room.

### 2.3. Case 3

A 54-year-old woman (weight, 54 kg; height, 153 cm) was scheduled to undergo conization for grade III cervical intraepithelial neoplasia. Her ASA score was II, as she was diagnosed with hypothyroidism. After obtaining written informed consent, we administered 0.2 mg glycopyrrolate intramuscularly. The patient’s initial vital signs were taken (BP 116/70 mmHg, HR 74 beats/min, RR 19 breaths/min, SpO2 100%). Supplemental oxygen was started, and it took 70 s for the patient to reach a MOAA/S score of two after kemimazolam induction. Her vital signs were stable, and her BIS score decreased to 74.

Afterward, 25 µg of fentanyl was administered, and 1 min later, the patient showed transient apnea, and oxygen saturation was reduced to 98%. Her breath was shortly resolved using jaw thrust and chin lift, and her respiratory rate recovered. There was no hypoxia (oxygen saturation <90%) and no requirement for masks or mechanical ventilation. Her BIS values ranged from 67 to 74, and there was no body movement throughout the procedure. The procedure ended 9 min after kemimazolam induction, and 14.6 mg of remimazolam was infused overall. Then, 6 min after discontinuation, the patient’s MOAA/S score increased to four, and she was transferred to the recovery room. She was discharged 5 min after entering the recovery room. She was completely amnestic and reported no side effects, including vivid dreams, nausea/vomiting, or pain.

### 2.4. Case 4

A 74-year-old woman (weight, 81 kg; height, 152 cm) with a suspected endometrial polyp was scheduled to undergo hysteroscopic endometrial polypectomy. She was previously diagnosed with stable angina, diabetes mellitus, and hypertension, and her ASA score was II. Written informed consent was obtained, and intramuscular glycopyrrolate (0.2 mg) was injected 30 min before the surgery. Her initial vital signs were as follows: BP, 180/69 mmHg; HR, 84 beats/min; RR, 20 breaths/min; and SpO2, 99%. Supplemental oxygen was administered before the induction of kemimazolam; 50 s after the bolus ketamine was applied and remimazolam was infused 6 mg/kg/h, the patient’s MOAA/S score reached 2, and the BIS value fell to 72. Shortly after, remimazolam was administered at 1 mg/kg/h for maintenance.

The vital signs at the time were as follows: BP, 165/52 mmHg; HR, 83 beats/min; RR, 20 breaths/min; and SpO2, 100%. Then, 25 µg of fentanyl was administered 4 min before the procedure, and the procedure commenced 8 min after induction. Her BP did not deviate beyond 20% from baseline, and other vital signs remained stable during the procedure. Her BIS values ranged from 68 to 75, and the MOAA/S score was below 3 throughout the surgery. No adverse events occurred until the end of the surgery, and remimazolam was discontinued. The surgery ended 31 min after induction, and a total of 48 mg remimazolam was injected. The MOAA/S score of the patient reached four after 8 min since remimazolam discontinuation. She did not remember anything about the procedure and reported a satisfactory experience. The modified Aldrete score of the patient met the discharge criteria 12 min after discontinuation, and no adverse events were observed until the patient left the recovery room.

## 3. Discussion

**Figure 1:**
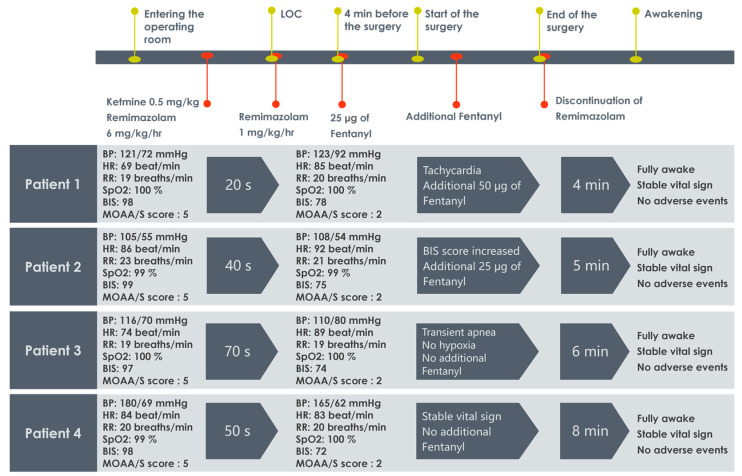
Timeline table and graph with vital signs and major events. LOC, loss of consciousness; BP, blood pressure; HR, heart rate; RR, respiration rate; SpO2, saturation of percutaneous oxygen; BIS, bispectral index; MOAA/S, modified observer assessment of alertness/sedation; sec, second; min, minute.

We conducted satisfactory monitored anesthesia care with kemimazolam in all four cases, and the timeline table and graph are presented below (Figure 1). A previous study compared remimazolam with propofol and infused remimazolam at a dose of 6 mg/kg/h for induction of general anesthesia [3]. The mean time to loss of consciousness (LOC) was 102.0 s, and the mean dose to LOC was 0.17 mg/kg. The mean time to LOC of propofol was 78.7 s. The mean time from the end of the study period to regaining consciousness was 14.9 min. The mean time to LOC in our study was 45 s (20, 40, 70, and 50 s each), and the mean dose to LOC was 0.075 mg/kg (0.033, 0.067, 0.117, and 0.083 mg/kg, respectively), which was even faster than propofol infusion. The mean time from remimazolam discontinuation to reaching a MOAA/S score of four was 5.75 min (4, 5, 6, and 8 min each) in our study. Kemimazolam led to a faster LOC than did remimazolam alone or propofol. The combination did not prolong sedation time, and it lowered the total induction dose of remimazolam.

Doi et al. revealed that 22% of patients who were administered remimazolam experienced hypotension and required vasopressors, and 6.3% required treatment for bradycardia [3]. None of the patients reported injection pain, and 7% and 6% experienced nausea and vomiting, respectively. Unlike in a previous study, hypotension and bradycardia were not observed in all four cases, from the induction of sedation to the end of surgery. Regarding other complications, none of the patients in our study reported injection pain at the time of induction or nausea/vomiting after the procedure, consistent with the previous study.

Pambianco et al. compared remimazolam and midazolam in patients undergoing colonoscopy [1]. They demonstrated that 3 out of 120 patients in the remimazolam group showed hypoxia (oxygen saturation <90%) and/or respiratory depression (respiratory rate <8 breaths per minute). Further, 2 of the 3 patients received 100 μg of fentanyl shortly before the respiratory events. None of the patients in our study showed hypoxia or respiratory depression after receiving an initial 25 μg of fentanyl or additional fentanyl. However, one patient showed transient apnea 1 min after administering the initial fentanyl.

Ketamine provides potent, dissociative anesthesia and analgesia, being a non-competitive NMDA receptor antagonist [4]. At lower doses, ketamine modulates opioid receptors by desensitizing central pain pathways. In a previous study, 1 μg/kg of fentanyl administration 4 min before surgery showed similar effects to remifentanil 0.05 μg/kg/min in monitored anesthesia care with propofol on recovery time, discharge time, and satisfaction scores [8]. In another study, patients were administered 0.5 mg/kg ketamine with propofol for induction, and pain scores for the first 3 h from the end of the operation were much lower compared to placebo [9]. We administered just 25 μg of fentanyl 4 min before the start of the procedure, considering the analgesic effect of ketamine, and two patients needed additional fentanyl (50 μg and 25 μg, respectively) intraoperatively. After the procedure, no additional opioid administration was needed, as none of the four patients reported severe pain. They did not request analgesia.

The main side effects limiting the use of ketamine during shorter procedures were recovery agitation and emergence symptoms. Both were more common at higher doses than at lower doses. Benzodiazepines are effective in reducing the incidence of emergence phenomena [4]. Sener et al. found that adding midazolam to ketamine can significantly reduce the incidence of recovery agitation, with the number needed to treat estimated at 6 of 90 [7]. Furthermore, adding midazolam improved patient satisfaction and did not significantly prolong sedation time or increase the incidence of other adverse events. This result corresponds to those of our study; we found no adverse events, such as vivid dreams, delirium, or emergence agitation.

Administering 0.5 mg/kg of ketamine is known to increase the BIS value, despite paradoxically deepening the level of hypnosis. BIS does not reflect the level of consciousness; it reflects cortical activity. Ketamine changes the EEG pattern, and these changes result in an increase in BIS levels independent of the depth of anesthesia [10]. Moreover, to date, the appropriate ranges of the BIS index for remimazolam anesthesia have not been fully clarified [2]. Thus, the infusion rate of remimazolam was adjusted only under the conditions where the BIS score was over 80 and the MOAA/S score exceeded 2, or where the BIS score was below 60 and the MOAA/S score reached 0 were simultaneously satisfied. Intraoperative awareness was evaluated throughout the procedure by checking the MOAA/S score, and the patients were asked about it after they were fully awakened. All four patients were totally amnestic and did not remember anything until the surgery was completed after receiving the remimazolam and ketamine combination.

## 4. Conclusions

The remimazolam and ketamine combination used for monitored anesthesia care has not been previously published. Kemimazolam may be an alternative to other agents or combinations used for monitored anesthesia care in brief gynecological procedures.

## Data Availability

Data are available only on request due to privacy/ethical restrictions.
